# A novel school-based intervention to improve nutrition knowledge in children: cluster randomised controlled trial

**DOI:** 10.1186/1471-2458-10-123

**Published:** 2010-03-10

**Authors:** Rajalakshmi R Lakshman, Stephen J Sharp, Ken K Ong, Nita G Forouhi

**Affiliations:** 1 MRC Epidemiology Unit, Institute of Metabolic Science, Cambridge, UK; 2 Department of Paediatrics, University of Cambridge, Cambridge, UK

## Abstract

**Background:**

Improving nutrition knowledge among children may help them to make healthier food choices. The aim of this study was to assess the effectiveness and acceptability of a novel educational intervention to increase nutrition knowledge among primary school children.

**Methods:**

We developed a card game 'Top Grub' and a 'healthy eating' curriculum for use in primary schools. Thirty-eight state primary schools comprising 2519 children in years 5 and 6 (aged 9-11 years) were recruited in a pragmatic cluster randomised controlled trial. The main outcome measures were change in nutrition knowledge scores, attitudes to healthy eating and acceptability of the intervention by children and teachers.

**Results:**

Twelve intervention and 13 control schools (comprising 1133 children) completed the trial. The main reason for non-completion was time pressure of the school curriculum. Mean total nutrition knowledge score increased by 1.1 in intervention (baseline to follow-up: 28.3 to 29.2) and 0.3 in control schools (27.3 to 27.6). Total nutrition knowledge score at follow-up, adjusted for baseline score, deprivation, and school size, was higher in intervention than in control schools (mean difference = 1.1; 95% CI: 0.05 to 2.16; p = 0.042). At follow-up, more children in the intervention schools said they 'are currently eating a healthy diet' (39.6%) or 'would try to eat a healthy diet' (35.7%) than in control schools (34.4% and 31.7% respectively; chi-square test p < 0.001). Most children (75.5%) enjoyed playing the game and teachers considered it a useful resource.

**Conclusions:**

The 'Top Grub' card game facilitated the enjoyable delivery of nutrition education in a sample of UK primary school age children. Further studies should determine whether improvements in nutrition knowledge are sustained and lead to changes in dietary behaviour.

## Background

In England, about 10% of children are obese, with a further 20-25% of children overweight [1]. Modelling estimates suggest that 40% of Britons are likely to be obese by 2025, and by 2050 Britain could be a mainly obese society [2]. The UK Government made a public service agreement target to 'reduce the proportion of overweight and obese children to 2000 levels by 2020' in the context of a broader strategy to tackle obesity as a whole'[3]. It is essential to find innovative ways of engaging the population in choosing healthier lifestyles [4,5].

Establishing healthy eating habits in young children may prevent various chronic health disorders in childhood and adult life, including obesity, diabetes, hypertension, cardiovascular disease, cancer and dental caries [6-8]. Schools provide an easily accessible setting for interventions targeted at children and parents to promote healthy lifestyles [9].

Nutrition education is important, though not sufficient to empower individuals to improve their diet. The Food and Health Action plan identified six key targets to improve the nation's diet, which include reduction of salt, sugar and saturated fat consumption and increased fibre and fruit and vegetable intake (Additional file 1) [10]. In order to achieve this it may be useful to increase knowledge of the nutritional content of commonly consumed foods [11].

The Department of Health has defined health related social marketing as 'the systematic application of marketing concepts and approaches to achieve behavioural goals relevant to improving health and reducing health inequalities' [12]. Using the principles of social marketing for health, we developed an educational intervention to increase children's nutrition knowledge by adapting a popular card game. We evaluated the effectiveness and acceptability of this intervention to improve nutrition knowledge in a cluster randomised controlled trial among primary school children in Cambridgeshire, UK.

## Methods

### Development of the intervention

The intervention comprised a card game 'Top Grub'^® ^developed by one of the authors (RL) and a package of classroom activities to teach the 'healthy eating' curriculum using these cards. This package was developed in collaboration with Cambridgeshire Personal, Social, Health Education (PSHE) service and Health Enterprise East (HEE). The card game 'Top Grub' was based on the popular children's card game 'Top Trumps'^®^.

**Figure 1:**
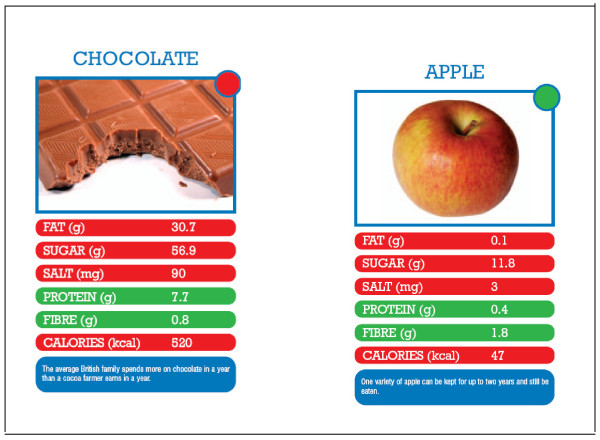
**Example of 'Top Grub' cards**.

Each 'Top Grub' card features one food item and gives its nutritional value in terms of fat, sugar, salt, protein, fibre and calories per 100 g, a picture, a fun fact about the food, and a red, amber or green dot based on the Food Standards Agency (FSA) recommendations for food profiling [13-15] (Figure 1). For red items the lower value is the better value and for green items the higher value is the better value. Two or more players play the game; each player holds their cards so that they can see the top card only. The aim of the game is to win all the cards. One person starts by calling out an item from their top card (e.g. fish fingers- protein 13.2 g). The other players then read out the value of the same item (i.e. protein) from their top card. Since protein is a green item, the player with the highest value wins and gets all the played cards including their own and keeps them at the bottom of their pile. If a red item is called (e.g. baked potato - sugar 1.2 g), the winner for the round is the person with the card that has the lowest value for that item on their top card. It is possible to incorporate other activities (such as picking out the cards with highest fat, salt, sugar contents and pointing out that chocolate has 300 times more fat, 5 times more sugar and 30 times more salt than an apple) and playing different versions of the game (details on http://www.topgrub.org.uk). The 33 cards cover popular food choices made by children of primary school age (based on a FSA survey) and 'healthier' alternatives were added to allow comparison, making it possible to understand why some foods are healthier than others (list available from corresponding author). The resource was adapted following piloting in three primary schools.

### Delivery of the intervention

At the start of the study, all the intervention schools received packs of the 'Top Grub' card game and the accompanying 'healthy eating' curriculum to be delivered during nine weeks of the summer term. This consisted of a number of classroom activities, for example- picking out the cards with the highest fat/sugar/salt content, discussing food labelling, placing the cards on the 'Balance of Good Health Plate' etc and teachers could pick and choose number and types of activities they wanted to use. Since this was a pragmatic trail and we wanted to assess if the intervention could be easily transferable with limited resources, no further input was provided. Teachers could use the cards and curriculum whenever time was available during the trial period and they were instructed to enable each child to take the card game home for a minimum of one weekend.

Schools in the control group used the existing 'healthy eating' curriculum. This included a wide variety of activities, such as 'healthy eating week', fruit tasting day, participation in 'healthy packed lunch box scheme', 'Active kids get cooking', development of whole-school healthy eating policies and use of recommended websites among others.

### Recruitment and randomisation of schools

Of the 208 state primary schools in Cambridgeshire, 205 (excluding the 3 pilot schools) were invited to take part in the trial. Only schools that were planning to deliver the 'healthy eating' curriculum for years 5 and 6 during the summer term of 2007 were asked to respond. Thirty-eight schools responded and entered the trial (comprising 2519 children in years 5 and 6).

Power calculations were based on the method described by Hayes and Bennett [16]. A pilot sample of 29 pupils from one school gave a mean (SD) for 'balanced diet' knowledge score of 14 (2.6) out of 20. Assumptions were an average of 50 eligible children in each school and a coefficient of variation of the true mean scores between matched pairs of schools of 0.25. Twenty schools in each arm would have provided 80% power to detect a difference of 4 points in mean balanced diet score between the intervention and control groups.

An independent statistician performed school-level randomisation within pairs of schools matched for deprivation (percentage children receiving free school meals) and school size (number of children in years 5 and 6). We matched the schools for deprivation and size as we felt that both these variables could have an effect on effectiveness of the intervention on the primary outcome, nutrition knowledge. In England, children who qualify for free school meals are from lower socio-economic backgrounds and other studies have used percentage of children eligible for free school meals as a proxy for deprivation.

### Development of the nutrition knowledge questionnaire

We performed a literature review for questionnaires on nutrition knowledge, attitudes and behaviour, but we found few that had been developed to evaluate specific school-based interventions to prevent obesity or to increase fruit and vegetable intake [17-29]. We contacted experts in the field to check for availability of a valid tool to assess nutrition knowledge. None of the questionnaires were specific for UK school children aged 9-11 years.

Using a combination of questions from the above sources, we developed a nutrition knowledge questionnaire. This was piloted among 29 children aged 9-11 years. The final modified questionnaire ('healthy eating quiz') comprised a total of 36 questions on nutrition knowledge and took around 15 minutes to complete. Fifteen of these questions tested knowledge of a balanced and healthy diet, five tested knowledge about the FSA 'Balance of Good Health Plate', fifteen tested ability to identify the healthiest option from a range of presented food items, and one question tested knowledge about the recommended number of fruit and vegetable portions.

At the start of the study all participating schools were sent the required number of baseline and follow-up questionnaires and instructions on how these were to be filled and returned at the end of the nine week period. Pre and post intervention questionnaires were printed on different coloured paper to minimize error.

Nutrition knowledge scores were calculated for three domains: Balanced diet, 'Balance of Good Health Plate' and Ability to identify healthier foods. The additional question on fruit and vegetable portions was included in the overall total score (maximum 36). Correct answers scored +1. Incorrect, blank and 'don't know' answers were scored zero points.

### Primary outcome measures

The primary outcome was nutrition knowledge at the end of the 9 week trial period as assessed in each child by the above questionnaire. Each child also completed the questionnaire at the beginning of the trial period, however in order to maintain confidentiality, most schools did not allow any identifiable information to be entered on the questionnaires and it was therefore not possible to calculate individual changes in nutrition knowledge score. Mean pre-trial and post-trial school score was used in the analysis to control for potential differences in baseline nutrition knowledge.

### Secondary outcome measures

In addition to knowledge, we sought to assess attitudes to healthy eating, at baseline and follow-up, using three questions (importance of eating a healthy diet, eating breakfast and drinking plenty of water throughout the day) that could be scored on a six point pictorial scale (from 'very important' to 'not important at all'). We also asked about whether they would try to eat a healthy diet with the options of -yes/no/maybe/don't know/ already eat a healthy diet.

In order to assess the acceptability of the intervention, children were asked by questionnaire how often they played the game, whom they played it with, how much they enjoyed it, and whether it helped them choose healthier foods. Further free text comments were invited. At the time of returning the completed questionnaires, teachers were encouraged to provide feedback on how useful they found the card game and curriculum as a resource.

### Data entry and Statistics

Responses to the questionnaires were double-entered onto a spreadsheet by an independent data-entry company blind to the school type (intervention/control). The mean nutrition knowledge scores at baseline and follow-up were calculated for each school. School-level mean scores at follow-up were compared between the intervention and control schools using linear regression (inversely weighted by the standard error of the school-level mean score), including mean baseline school score, deprivation and school size as covariates.

Two of the control schools inadvertently used the cards during the trial period. Analyses were performed on an intention to treat basis in order to preserve randomisation and avoid bias. A sensitivity analysis excluding those two schools gave substantially similar results. Analyses were performed using STATA version 9.2 (STATACORP, College Station, Texas, USA).

### Ethical Approval and consent

As the study assessed a new curriculum and change in nutrition knowledge with no identifiable data or anthropometry measurements, ethical approval was not required.

The head teachers of participating schools filled out a reply slip and gave consent for participation. No individual student or teacher consent was obtained.

## Results

**Figure 2:**
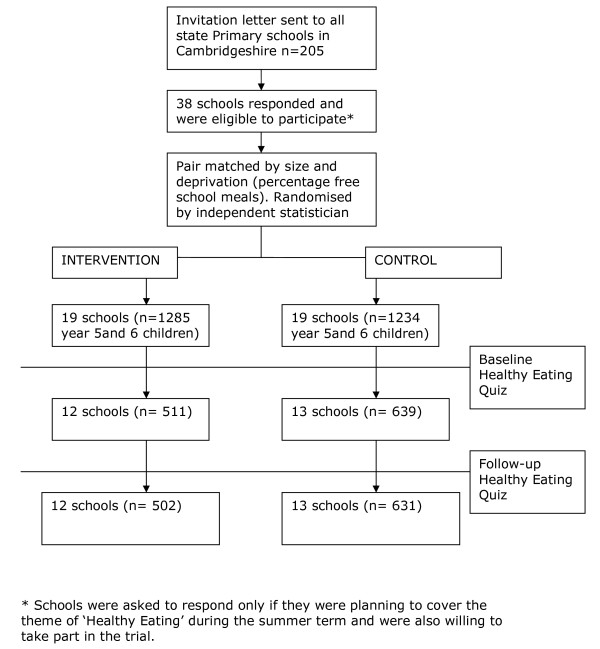
**Progress of schools and pupils through the trial**.

Twelve intervention schools and 13 control schools (comprising 502 and 631 children respectively) completed the trial and returned follow-up questionnaires (Figure 2). For these schools, school size (number of children in years 5 and 6) was similar in the intervention group (median: 57; range 12 to 166) and the control group (50; 18 to 166). Percentage of children receiving free school meals was also similar in intervention schools (median: 7%; range: 1 to 25%) and control schools (6%; 0 to 31%) (Table 1). Nutrition knowledge scores were high at baseline in both intervention and control schools with scores being higher in the intervention schools (28.3/36) compared to control schools (27.3/36).

**Table 1:** Characteristics of schools in Intervention and Control groups

School id	Group	Deprivation	Size	Mean baseline score	Mean follow-up score	Difference
1	Intervention	9	124	27.4	28.1	0.7
2	Intervention	6	120	28.6	29.1	0.5
3	Intervention	5	38	29.0	29.0	0.0
4	Intervention	20	12	26.3	29.0	2.6
5	Intervention	4	25	30.0	28.3	-1.8
6	Intervention	4	166	29.2	29.0	-0.1
7	Intervention	25	88	29.4	28.8	-0.6
8	Intervention	16	58	28.3	31.0	2.7
9	Intervention	1	70	27.6	29.1	1.5
10	Intervention	3	57	26.6	29.7	3.2
11	Intervention	12	35	29.1	30.2	1.1
12	Intervention	8	42	28.2	29.0	0.8

13	Control	6	104	26.2	28.0	1.8
14	Control	0	34	30.7	28.7	-2.0
15	Control	4	41	30.2	30.5	0.3
16	Control	7	23	23.9	28.3	4.3
17	Control	3	18	27.2	26.1	-1.1
18	Control	2	56	28.9	30.6	1.6
19	Control	7	108	27.0	27.3	0.2
20	Control	0	109	28.0	28.4	0.4
21	Control	3	28	27.2	28.9	1.7
22	Control	31	49	23.7	23.0	-0.8
23	Control	19	50	27.8	25.7	-2.1
24	Control	8	84	26.8	25.6	-1.1
25	Control	8	166	26.9	27.7	0.9

Deprivation measured as percentage of children receiving free school meals

Only six of the school pairs completed the study. The main reason given for non-completion was time pressure of the school curriculum. The schools that did not complete the trial had similar levels of deprivation and school size as schools that completed the study (median percentage of children receiving free school meals = 7%; range: 0 to 25% and median number of children in years 5 and 6 = 49; 16 to 154).

Five hundred and thirty children answered that they had played the card game. Median number of occasions was 4 (range 1 to 40). More children reported playing the game in intervention schools (89%) than control schools (13% children in two schools). Children reported playing the game with friends (n = 524) parents (n = 63), siblings (n = 50) and others (n = 81).

### Total Nutrition Knowledge Score

Mean total nutrition knowledge score increased in both intervention (baseline to follow-up: 28.3 to 29.2) and control schools (27.3 to 27.6). Total nutrition knowledge score at follow-up, adjusted for baseline score, deprivation and school size; also weighted for school size, was higher in intervention than in control schools (mean difference = 1.1; 95% CI: 0.05 to 2.16; p = 0.042; Table 2).

**Table 2:** Mean and SD of the school-level mean scores for total nutrition knowledge, and its sub-domains, at baseline and at follow-up in the intervention and control schools.

	Baseline		Follow-up			
	**Intervention n = 12**	**Control n = 13**	**Intervention n = 12**	**Control n = 13**	*Estimated difference at follow up (95% CI)	*p value

Total number of pupils	511	639	502	631		

Total score (max. 36)	28.3(1.1)	27.3(2.0)	29.2(0.8)	27.6(2.1)	1.1(0.05 to 2.16)	*0.042*

Balanced diet domain (max. 15)	11.6(0.4)	11.3(0.9)	12.1(0.5)	11.5(0.9)	0.6(0.1 to 1.1)	*0.018*
Balance of Good Health Plate domain (max. 5)	3.7(0.4)	3.5(0.4)	4.1(0.4)	3.6(0.3)	0.3(0.0 to 0.6)	*0.041*
Ability to identify healthier foods (max. 15)	12.1(0.6)	11.6(0.9)	12.1(0.4)	11.6(1.0)	0.3(-0.3 to 0.8)	*0.375*

* p value and estimated difference in mean score between intervention and control schools at follow-up was adjusted for deprivation (percentage of children receiving free school meals), school size, and baseline score; also weighted for school size.

#### Healthy/Balanced diet domain

Mean scores in this domain increased in both intervention (baseline to follow-up: 11.6 to 12.1) and control schools (11.3 to 11.5; Table 2). Mean Healthy/Balanced diet domain score at follow-up, adjusted for baseline score, was higher in intervention than in control schools (mean difference = 0.6; 95% CI: 0.1 to 1.1; p = 0.018).

#### Balance of Good Health Plate domain (currently FSA recommended Eatwell plate)

Mean scores in this domain increased in both intervention (baseline to follow-up: 3.7 to 4.1) and control schools (3.5 to 3.6; Table 2). Mean Balance of Good Health Plate domain score at follow-up, adjusted for baseline score, tended to be higher in intervention than in control schools (mean difference = 0.3; 95% CI: 0.0 to 0.6; p = 0.041).

#### Ability to identify healthier foods

Mean scores in this domain did not increase in either intervention or control schools (Table 2).

### Attitudes to healthy eating

Over 95% of children answered that eating breakfast and drinking plenty of water throughout the day was 'very important' or 'important' and could give good reasons for this. More than 90% answered it was 'very important' or 'important' to eat a healthy diet. At follow-up, more children in the intervention schools said they 'are currently eating a healthy diet' (39.6%) or 'would try to eat a healthy diet' (35.7%) than in control schools (34.4% and 31.7% respectively; chi-square test p < 0.001), although this may have been due to baseline differences.

### Acceptability of the intervention

Over 75% of the 530 children who played the card game answered that they enjoyed it and 70% considered that the game enabled them to choose healthier foods. Of the 198 free text comments about the game almost all were positive or constructive (Additional file 2). Two children reported that it was 'boring', two reported it was 'not very good' and one reported 'did not learn much'. Only three teachers provided written feedback (Additional file 2) but informal feedback to Cambridgeshire PSHE service was positive and the resource has been incorporated into the 'Lunchbox Challenge' package of activities used to promote healthy eating across all primary schools in Cambridgeshire.

## Discussion

This pragmatic cluster randomised controlled trial showed that a novel, educational intervention, delivered without additional teacher training or professional input, achieved a modest increase in nutrition knowledge in a sample of UK primary school children, who found the intervention to be enjoyable and engaging. While nutrition knowledge scores increased, ability to identify healthier foods did not improve.

### Comparison with other multi-component school-based interventions

There have been two recent systematic reviews on school-based interventions to promote healthier lifestyles and prevent obesity in children [30,31]. The Swedish Council on Technology Assessment in Health Care identified 39 studies, of which 15 were positive, 24 were neutral, and none showed negative results. Those authors concluded that school-based programmes that combine the promotion of healthy dietary habits and physical activity may be effective in preventing childhood obesity [30]. A Cochrane review of 19 school-based studies concluded that diet and exercise interventions could promote a healthy diet and increase physical activity levels, but were not effective in preventing weight gain [31]. Studies varied in the duration and intensity of intervention, whether they targeted both physical activity and nutrition behaviours [27-29] or focussed on only one dimension such as TV watching [32] or restricting drinking of carbonated drinks [33]. There is still uncertainty as to the components of an effective intervention [34]. More recent studies suggest that higher intervention dose and longer duration increase effectiveness. For example, the Stockholm Obesity Prevention Programme (STOPP), which since 2001 has aimed to reduce the intake of fat and sugar in schools (ban on sweets, buns and sweetened drinks) showed that prevalence of overweight or obesity decreased in the intervention schools and increased in the control schools [35,36]. Similarly the Harvard School of Public Health 5-2-1-Go! Program (eat 5 servings of fruit and vegetables daily, limit screen time to no more than 2 hours a day, and get at least 1 hour of physical activity daily) that includes the 'Planet Health' curriculum has shown promising results over many years [9,37]. On the other hand school programmes with shorter durations of around one year, such as the Christchurch Obesity Prevention Programme in Schools (CHOPPS) [38] and the Active Programme Promoting Lifestyles in Schools (APPLES), have not been successful [33,39]. We acknowledge that we did not assess behaviour change or physical measures of adiposity. While increasing nutrition knowledge may be an important initial step, ensuring translation to behaviour change will likely require a more intensive intervention and more complex evaluation [40].

### Comparison with other school-based nutrition education programmes

There are no studies similar to ours and only a few studies incorporating nutrition education as a single component. A study evaluating the Food and Agriculture Organisation's global school-based nutrition education initiative 'Feeding Minds, Fighting Hunger', randomised 670 children from 10 schools, in grades 8 and 9, and similar to our study found that nutrition knowledge improved in the intervention and control groups with the effect size being larger in the intervention schools [41]. The 'Michigan Model Nutrition Curriculum' was evaluated among 576 children. The study showed that nutrition knowledge improved in the intervention group and students in the intervention group were more likely to eat fruits and vegetables and less likely to eat junk food than the control group [42]. A study of the effect of the nutrition education program 'Colour My Pyramid' http://www.MyPyramid.gov consisting of six classes taught over a 3-month period, showed that nutrition knowledge only increased in the control group, and there were no significant differences in BMI percentiles [43].

A 'Traffic Light Nutrition Tool' was evaluated among 69 children aged 5-7 years in one primary school in UK, using a non-randomised, pre- and post-test design. This study showed that knowledge improved and children's refusing behaviour for 'red food' items increased. Positive attitude scores and asking behaviour for both red and green food items decreased [44]. The 'Nutrition for Life' programme was evaluated among 1863, 7^th ^and 8^th ^grade students, and showed modest but significant differences in nutrition attitude, behaviour and knowledge scores post-intervention [45]. Another study evaluated the effect of a 'Food Guide Pyramid' lesson on nutrition knowledge among 15 children aged 9-12 years in a refugee after-school programme. A 12-item knowledge questionnaire was used and mean knowledge scores did not increase significantly from pre-to post-test, however, scores that measured ability to identify food groups and the number of servings for food groups increased, while scores on the ability to identify the importance of each food group for health decreased [46].

### Strengths and Limitations

Effective school-based interventions need to be long term and easily sustainable. For situations of limited resources, we need to develop interventions that do not rely on continued input from health professionals and fit in with the education curriculum. We have successfully developed and tested such an intervention with input from children, teachers and dieticians. The primary outcome in our study was change in nutrition knowledge and since we did not find any existing tool that could be used to assess nutrition knowledge in this age group, we developed our own tool. The reliability and validity of this tool needs to be tested before it can be used in other studies.

A major limitation of our study was that, due to issues of confidentiality raised by several of the schools, individual-level data could not be matched at baseline and follow-up and we had to use aggregate school-level data to compare scores in the intervention and control schools. Since baseline scores were higher in intervention schools compared to control schools, we cannot rule out the possibility that the statistically significant difference in follow-up scores could have been due to inadequate adjustment for baseline scores. Ethnicity and social class of each individual were not available, and hence we could not adjust for them in the analysis.

We recognize that our sample of schools had lower levels of deprivation, as assessed by number of children receiving free school meals (6 to 7%) compared to 16.9% for all primary schools in England in 2005 [47]. Indeed baseline nutritional knowledge was already high and the potential effect size of our intervention could be larger in areas of greater deprivation or in children with lower baseline nutrition knowledge, as other studies have suggesting a 'ceiling' effect in children's nutrition knowledge [29,48]. We did not collect any information on process measures and schools may have varied in the delivery of the intervention and completion of questionnaires. Two of the control schools inadvertently used the card game during the trial period. We followed an intention to treat analysis protocol, however a sensitivity analysis without these two schools showed similar results.

## Conclusions

In conclusion, this trial shows that baseline knowledge and intentions about a healthy diet are good among primary school children aged 9-11 years. Although we found a modest increase in nutrition knowledge scores, further studies are needed to assess whether this intervention can form part of a more complex-behaviour change intervention to prevent childhood obesity, since change in knowledge alone is unlikely to result in behaviour change. We believe that public health interventions will have to be multi-component, long-term, sustainable without extra professional input, use novel approaches and modification of the 'obesogenic environment' in order to halt or reverse the tide of increasing obesity.

## Abbreviations

FSA: Food Standards Agency; PSHE: Personal, Social and Health Education

## Competing interests

The authors declare that they have no competing interests.

## Authors' contributions

All authors contributed to the development of the protocol and outcome, and to writing the paper. RL performed the statistical analysis with SS and wrote the first draft of the paper. All authors read and approved the final manuscript.

## Funding

Health Enterprise East, NHS innovations hub for East of England supported the production of the card game and the revised curriculum, and organised their distribution. The MRC Epidemiology Unit supported data entry of the questionnaires.

## Pre-publication history

The pre-publication history for this paper can be accessed here:

http://www.biomedcentral.com/1471-2458/10/123/prepub

## Supplementary Material

Additional file 1 Targets from National Food and Health Action Plan. Click here for file

Additional file 2 Examples of comments from children and teachers. Click here for file
