# Supplementary material for: A novel school-based intervention to improve nutrition knowledge in children: cluster randomised controlled trial
Source: BMC Public Health. 2010 Mar 10;10:123. doi: 10.1186/1471-2458-10-123 (PMC2847978; doi:10.1186/1471-2458-10-123)
Supplement: Additional file 1 — Targets from National Food and Health Action Plan. [file 1471-2458-10-123-S1.DOC]

**Additional file 1: Targets from National Food and Health Action Plan**

- Increase the average consumption of a variety of fruit and vegetables to at least five portions per day (currently 2.8 portions per day);
- Increase the average intake of dietary fibre to 18 grams per day (currently 13.8 grams per day);
- Reduce the average intake of salt to 6 grams per day by 2010 (currently 9.5 grams per day);
- Reduce the average intake of saturated fat to 11% of food energy (currently 13.3%);
- Maintain the current trend in the average total intake of fat at 35% of food energy (currently 35.3%); and
- Reduce the average intake of added sugar to 11% of food energy (currently 12.7%).
