# Supplementary material for: A novel school-based intervention to improve nutrition knowledge in children: cluster randomised controlled trial
Source: BMC Public Health. 2010 Mar 10;10:123. doi: 10.1186/1471-2458-10-123 (PMC2847978; doi:10.1186/1471-2458-10-123)
Supplement: Additional file 2 — Examples of comments from children and teachers. [file 1471-2458-10-123-S2.DOC]

**Additional file 2: Examples of comments from children and teachers**

**Children’s comments**

- The game makes you learn with entertainment
- I think it made me realise how much fat, salt and sugars in food
- It was cool loved it
- It is a great game
- It’s the best card game ever
- Top Grub helped me find out what the ratings for different foods are
- I think Top Grub told me things I did not know before
- I think the game is excellent and will help children learn about what they are eating
- My mum is cutting down on salt massively, so now I can tell her what stuff she can have without there being salt
- I think it shows us really what we eat
- I liked the game because now I am food wise. It was fab. Star rating 5 stars
- Playing the game was better than having a normal science lesson
- The game style could be a lot more interesting
- Make it bolder and brighter

**Teacher’s comments**

- The children have thoroughly enjoyed and I hope that your research will show that they have also learnt a lot too.
- It was a great activity. The children love ‘Top Trumps' and they understood what to do straight away. Thanks for them. Hope they have learnt from them and the other activities we’ve done.
- I thought they were excellent. I will be delivering the same programme next year. I think it has been well thought out and hopefully will make a real difference to my pupils.
